# Electric-Field-Treated Ni/Co_3_O_4_ Film as High-Performance Bifunctional Electrocatalysts for Efficient Overall Water Splitting

**DOI:** 10.1007/s40820-022-00889-3

**Published:** 2022-07-22

**Authors:** Junming Li, Jun Li, Jun Ren, Hong Hong, Dongxue Liu, Lizhe Liu, Dunhui Wang

**Affiliations:** 1 National Laboratory of Solid State Microstructures, Collaborative Innovation Center of Advanced Microstructures, Jiangsu Provincial Key Laboratory for Nanotechnology, School of Physics, Nanjing University, Nanjing, 210093 People’s Republic of China; 2 Hangzhou Dianzi University, Hangzhou, 310018 People’s Republic of China

**Keywords:** Bifunctional electrocatalyst, Overall water splitting, Co_3_O_4_ film, Oxygen vacancies, Electric field

## Abstract

**Highlights:**

- A novel physical approach is proposed to enhance the electrocatalytic performance by electric field.
- Under the action of electric field, some stable conductive filaments consisting of oxygen vacancies are formed in the Ni/Co_3_O_4_ film, which remarkably reduces the system resistivity.
- The electric-field-treated Ni/Co_3_O_4_ material exhibits significantly superior activity and stability as a bifunctional electrocatalyst for overall water splitting, and its performance exceeds the state-of-the-art electrocatalysts.

**Abstract:**

Rational design of bifunctional electrocatalysts for oxygen evolution reaction (OER) and hydrogen evolution reaction (HER) with excellent activity and stability is of great significance, since overall water splitting is a promising technology for sustainable conversion of clean energy. However, most electrocatalysts do not simultaneously possess optimal HER/OER activities and their electrical conductivities are intrinsically low, which limit the development of overall water splitting. In this paper, a strategy of electric field treatment is proposed and applied to Ni/Co_3_O_4_ film to develop a novel bifunctional electrocatalyst. After treated by electric field, the conductive channels consisting of oxygen vacancies are formed in the Co_3_O_4_ film, which remarkably reduces the resistance of the system by almost 2 × 10^4^ times. Meanwhile, the surface Ni metal electrode is partially oxidized to nickel oxide, which enhances the catalytic activity. The electric-field-treated Ni/Co_3_O_4_ material exhibits super outstanding performance of HER, OER, and overall water splitting, and the catalytic activity is significantly superior to the state-of-the-art noble metal catalysts (Pt/C, RuO_2_, and RuO_2_ ǁ Pt/C couple). This work provides an effective and feasible method for the development of novel and efficient bifunctional electrocatalyst, which is also promising for wide use in the field of catalysis.
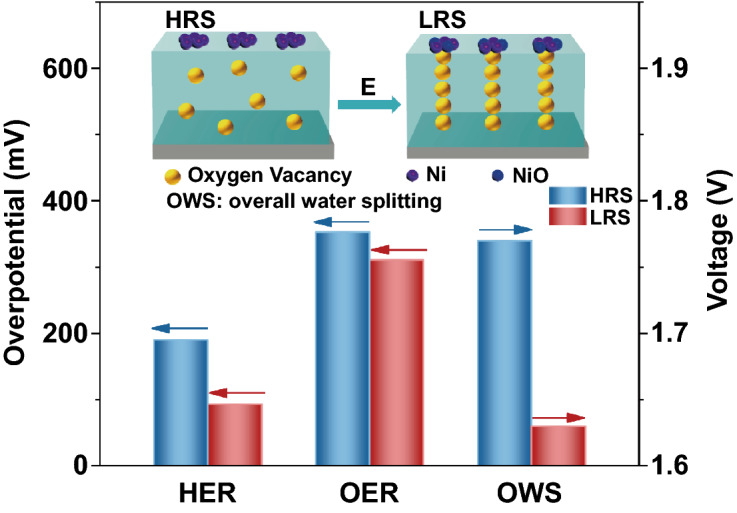

**Supplementary Information:**

The online version contains supplementary material available at 10.1007/s40820-022-00889-3.

## Introduction

The continuous and rapid consumption of fossil fuels and the resulting environmental problems have brought tremendous pressure to the sustainable development of modern society [1, 2]. Therefore, the development of sustainable clean energy such as hydrogen is increasingly urgent due to the clean, efficient and renewable advantages [3, 4]. Electrochemical water splitting is a major research area that attracts a widespread attention in order to reduce the dependence on traditional fossil energy and clean up the environment [5–7]. However, the electrocatalytic efficiency is still unsatisfactory due to the inevitable dynamic overpotential in hydrogen evolution reaction (HER) and oxygen evolution reaction (OER) processes [8]. Thus, it is of great significance to make efforts to reduce the overpotential of catalytic reaction. At present, the most active catalysts for HER and OER are the noble metal-based materials (Pt, Ir, Ru, etc.), which are expensive, scarce in reserves, and difficult to realize large-scale application. Hence, it is of great significance to exploit efficient, low-cost, and abundant electrocatalysts with high activity for HER and OER.

Overall water splitting which includes the process of both HER and OER has received extensive attention due to its simplified setups, avoiding the production of different electrocatalysts and reducing the costs [9, 10]. However, the HER and OER catalysts generally react in different electrolyte medias with unmatched pH ranges [11, 12]. In general, non-noble metal-based HER catalysts (e.g., chalcogenides, phosphides, and carbides) exhibit excellent catalytic activity in acidic media, but the OER catalysts (e.g., transition metal oxides and (oxy)hydroxides) show excellent catalytic activity in alkaline electrolytes [13]. When the two-electrode reactions are paired together in an electrolytic cell with the same electrolyte, the integration is incompatible and the catalytic performance is mediocre [14]. Therefore, it keeps a challenge to construct a single bifunctional catalyst with excellent HER and OER activities in the same electrolyte. Tremendous efforts have been devoted to developing bifunctional electrocatalysts, for instance, utilizing the synergistic effects of different species to construct multi-component catalysts [15, 16], doping heteroatoms [17, 18], or introducing defects [19, 20] into electrocatalysts. Recently, some bifunctional electrocatalysts have been developed and applied to overall water splitting in alkaline solutions, such as transition metal oxides (e.g., MoO_2_, NiCoO_4_, Co_3_O_4_) [21–23], sulfides (e.g., NiCo_2_S_4_) [24], selenides (e.g., NiSe) [25], phosphates (e.g., Ni_5_P_4_) [26], and layered double hydroxides (e.g., NiFe LDHs) [27]. However, the low conductivity of these materials has hindered their practical application as bifunctional electrocatalysts [11].

**Fig. 1:**
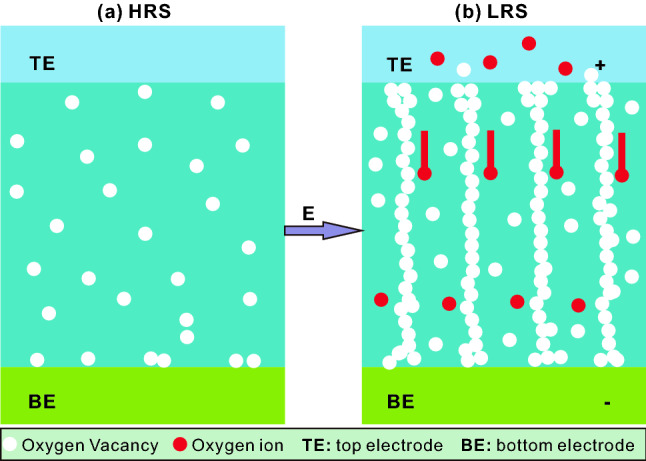
Schematic diagram of resistance switching (RS) mechanism: **a** high resistance state (HRS); **b** low resistance state (LRS)

The oxide materials usually possess a certain amount of oxygen vacancies (OVs), and after treated by an electric field, some conductive channels can be formed in these oxides by aligning the OVs, converting the system from a high resistance state (HRS) to a low resistance state (LRS) [28, 29]. This phenomenon is called as the resistance switching (RS) effect, which has been widely observed in various oxide semiconductors sandwiched by two metal electrodes [30, 31]. The schematic diagram of RS mechanism is shown in Fig. 1. In the case of the initial state, there are some OVs scattered in the oxide semiconductor film, which makes the device show a HRS (Fig. 1a). When a voltage is applied on the device, the oxygen ions in the oxide move toward the anode along the direction of electric field and gradually forms the conductive filaments (i.e., conductive channels) consisting of OVs. Once the conductive filaments penetrate the oxide film, the device immediately converts to the LRS, which is shown in Fig. 1b. Since the conductive filaments consisting of OVs are constructed in the LRS film, the electron migration can be accelerated which is beneficial to improve the catalytic activity [32].

Spinel Co_3_O_4_ is a promising candidate to substitute noble metal-based electrocatalysts for overall water splitting, owing to low price, outstanding redox capability, and relatively good anticorrosion performance in alkaline environment [33, 34]. Unfortunately, single-component oxide exhibits intrinsically low electrical conductivity and unfavorable hydrogen adsorption/desorption capability, which hinders its large-scale application as a bifunctional electrocatalyst in alkaline media [35, 36]. As we know, Co_3_O_4_ is a well-studied RS material, which provides a good opportunity to investigate the influence of RS effect on the electrocatalytic performance [37, 38]. In addition, it is reported that Ni metal has a suitable H atom binding energy close to Pt [39, 40]. Thus in this paper, Co_3_O_4_ film with Ni metal electrodes is prepared to study the influence of RS effect on electrocatalysis. Unlike traditional microstructural strategies, this work concentrates on an easy and practical solution to improve electrocatalytic performance via external electric field treatment. Based on the RS mechanism, a large number of conductive filaments consisting of OVs are formed in the Co_3_O_4_ film after treated by an electric field, which reduces the resistance of the Co_3_O_4_ film by almost 2 × 10^4^ times. The high conductivity of catalyst material endows the fast charge transport capability between the catalyst surface and the conductive support, which is beneficial to improve the catalytic activity [41]. As expected, the LRS sample possesses excellent HER and OER performance. Moreover, the conductive filaments consisting of OVs can be preserved in bulk for a long time, which ensures super outstanding stability of the system. More importantly, when integrated together, the LRS ǁ LRS couple exhibits superior performance to the noble metal Pt/C ǁ RuO_2_ electrocatalyst for overall water splitting.

## Experimental Section

### Preparation of Ni/Co_3_O_4_/Pt Device

Co_3_O_4_ film was deposited on Pt/SiO_2_/Si substrate using pulsed laser deposition (PLD) with a 248 nm KrF excimer laser at 3 Hz. The energy laser fluence was kept 2 J cm^−2^ by using a constant energy mode. The target material has a stoichiometric ratio, and the base vacuum of the chamber was lower than 5.0 × 10^–6^ Pa prior to deposition. The oxygen pressure was maintained at 10 Pa and the substrate temperature was 500 °C during the deposition of Co_3_O_4_ film. After deposition, the film was annealed in situ at 500 °C for 30 min. Circular Ni top electrodes with diameter of 100 μm were deposited on Co_3_O_4_ film with a shadow mask using the same PLD. The target material is a Ni target with the purity larger than 99.99%, and the base vacuum was below 5.0 × 10^–6^ Pa. The Ni top electrodes were deposited under vacuum pressure of 10^–3^ Pa at room temperature.

### Material Characteristics

Powder X-ray diffraction (XRD) patterns were collected on a Bruker D8 Advance powder diffractometer (Cu Kα, td-3500, Tongda). Sample morphology, thickness, and element analysis of the films were characterized by field emission scanning electron microscopy (FESEM) with an energy dispersive spectrometer (GeminiSEM 500, ZEISS, Germany). X-ray photoelectron spectroscopy (XPS) was detected on a Phi5000 VersaProbe (ULVAC-PHI, Japan) using 200 W monochromated Al Kα radiation as the X-ray source, and binding energies were calibrated against the C 1* s* signal at 284.60 eV of adventitious hydrocarbons. The electrical properties of the samples were recorded by Keithley-2410 m with a dual probe configuration. The electron paramagnetic resonance (EPR) spectra were carried out by Bruker A300 spectrometer. Raman spectra were obtained by Renishaw inVia with an excitation laser source of 532 nm.

### Electrochemical Measurements

Electrochemical measurements were carried out on an electrochemical workstation (CHI760E) equipped with a standard three-electrode electrochemical cell. The as-prepared electrocatalysts was used as working electrode. The Hg/HgO and graphite rod were served as the reference electrode and counter electrode, respectively. All measured potentials were converted to a reversible hydrogen electrode (RHE) scale based on the equation: *E* (vs. RHE) = *E* (vs. Hg/HgO) + 0.059 × pH + 0.098 V. A KOH solution (1 M) was employed as the electrolyte, and the linear sweep voltammetry curves were measured at a scan rate of 10 mV s^−1^. All linear sweep voltammetry (LSV) curves were corrected with iR compensation using the equation: *E*_iR-corrected_ = *E* − *iR*, where *E* is the original potential, *R* is the solution resistance, *i* is the corresponding current, and *E*_iR-corrected_ is the *iR*-corrected potential.

The electrochemical impedance spectroscopy (EIS) was carried out at an open circuit potential from 0.1 to 10^5^ Hz with AC amplitude of 5 mV. The electrochemically effective surface area (ECSA) was estimated by electrochemical double-layer capacitances (*C*_dl_). The *C*_dl_ was determined with cyclic voltammetry (CV) measurements without obvious electrochemical reactions at various scan rates of 20, 40, 60, 80, and 100 mV s^−1^ in the potential range of 0.18–0.26 V vs. RHE. During the electrochemical test, the area of the working electrode immersed in the electrolyte was 0.2 cm^2^ to ensure that the electrode immersed in the electrolyte has a large number of conductive filaments.

### DFT Calculation

All theoretical calculations were performed by Vienna ab initio simulation package (VASP), with the generalized gradient approximation (GGA) and Perdew–Burke–Ernzerhof (PBE) method. The atomic forces were converged within 0.01 eV Å^−1^, which had a good convergence. Monkhorst–Pack k-points grid was 7 × 7 × 1 and the plane-wave cutoff energy was set to 460 eV for the structure optimization.

## Results and Discussion

### Composition and Structure of Device

**Fig. 2:**
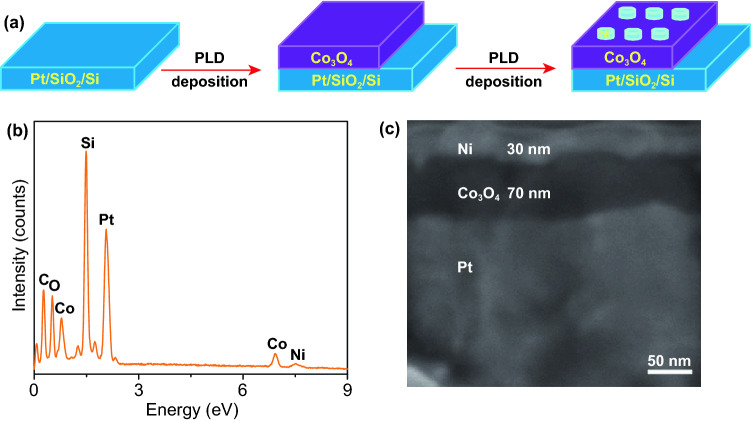
**a** Schematic image of the formation process. **b** Element analysis of the prepared device. **c** SEM images of the cross section of the Ni/Co_3_O_4_/Pt device

Figure 2a shows the preparation process of the Ni/Co_3_O_4_/Pt/SiO_2_/Si device using PLD. Co_3_O_4_ film is deposited onto a Pt/SiO_2_/Si substrate, and subsequently, Ni circular electrodes are deposited on Co_3_O_4_ film with a shadow mask. Here, Ni and Pt electrodes act as the top and bottom electrodes, respectively, which are utilized for carrying out the RS operation. The element analysis of the prepared device is performed by the energy dispersive X-ray (EDX) spectrum. The EDX spectrum demonstrates that the device is composed of Co, Ni, O, C, Pt, and Si elements (Fig. 2b), in which the Si and Pt elements come from the substrate and the C element comes from the environment. The surface and cross section structure of the device are investigated by FESEM. The Ni circular electrodes with a diameter of about 100 µm are observed on the surface of the Co_3_O_4_ film in the surface image of Ni/Co_3_O_4_/Pt device (Fig. S1). Moreover, in the cross-sectional SEM image of the device with Ni electrode, the thicknesses of Co_3_O_4_ film and Ni electrode are estimated to be 70 and 30 nm, respectively (Fig. 2c).

### Characteristic of RS Effect

**Fig. 3:**
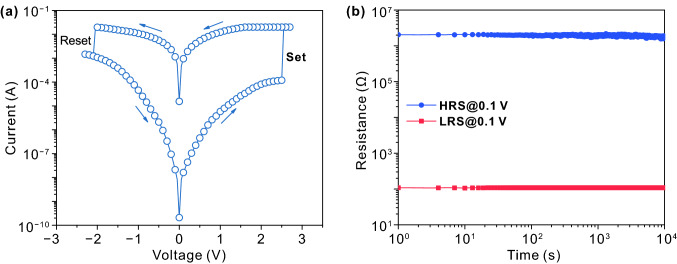
**a** *I*-*V* curve and **b** retention properties of the RS device. The current compliance (*I*_cc_) is 0.02 A

To verify the RS effect in Co_3_O_4_ film, current–voltage (*I-V*) sweep voltammetry is performed and the corresponding result is shown in Fig. 3a. Before the measurement, an electroforming process with a voltage of 4.3 V is required to switch the Co_3_O_4_ film from HRS to LRS by aligning OV along the electric field direction to form OV conductive filaments (Fig. S2). When a negative sweeping voltage is applied to the device, the current flow maintains at a high level owing to the nonvolatile characteristic of conductive filaments, and then shows a sharp drop at -2.1 V, which corresponds to an abrupt change from LRS to HRS. By sweeping the voltage reversely, the device switches from HRS to LRS with the current jumping suddenly at 2.5 V, demonstrating typical bipolar resistive switching (BRS) behavior [38, 42]. To further evaluate the stability of RS behavior, the time-dependent resistance characteristics are measured on the HRS and LRS of device. As shown in Fig. 3b, the retention time is measured up to 10^4^ s without any obvious degradation, indicating the extraordinary retention performance of the device. It is worth mentioning that the resistance ratio of HRS sample to LRS sample is almost 2 × 10^4^, implying that the device of LRS possesses high conductivity and has potentials for showing excellent electrocatalytic performance.

### Structure and Oxygen Vacancy Characterization of the Samples

**Fig. 4:**
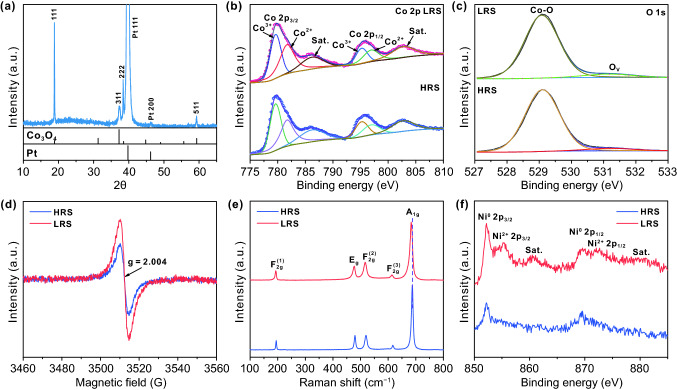
**a** XRD pattern of the device. XPS spectra at **b** Co 2*p* level and **c** O 1*s* level. **d** EPR spectra. **e** Raman spectra with the excitation line at 532 nm. **f** XPS spectra at Ni 2*p* level on the surface of LRS and HRS samples

Figure 4a shows the XRD pattern of the prepared device. In addition to the diffraction peaks of the substrate, the peaks located at 19.0°, 36.9°, 38.5°, and 59.4° can be ascribed to (111), (311), (222), and (511) planes of Co_3_O_4_ (JCPDS No. 43-1003), respectively, demonstrating that the pure phase nature of Co_3_O_4_ film is performed to analyze the chemical element and OVs of the prepared catalysts. The XPS spectra of Co 2*p* and O 1*s* levels in both LRS and HRS samples are shown in Fig. 4b–c. These spectra are measured after removing top Ni electrodes by Ar^+^ sputtering with the negligible Ar^+^ ion implantation and corresponding damage [43]. As shown in Fig. 4b, the Co 2*p* XPS spectra exhibit two sharp peaks of 2*p*_1/2_ and 2*p*_3/2_ and a pair of weak satellite peaks, demonstrating the coexistence of Co^2+^ and Co^3+^ state in the pure Co_3_O_4_ films [44, 45]. From the fitted curve of Co 2*p* region, the peaks at the binding energies of 781.7 and 796.9 eV can be attributed to Co^2+^ 2*p*_3/2_ and Co^2+^ 2*p*_1/2_, respectively. The satellite peaks locate at 786.2 and 802.7 eV, respectively. Additionally, the peaks around 779.6 and 795.2 eV at the lower energy sides correspond to Co^3+^ 2*p*_3/2_ and Co^3+^ 2*p*_1/2_, respectively. Moreover, the rate of Co^3+^/Co^2+^ in LRS sample (0.85) is lower than that of HRS sample (1.06), suggesting that more low-valence Co (Co^2+^) ions exist in the LRS sample. According to previous reports, the reduction of Co^3+^ ions to Co^2+^ ions is usually accompanied by the generation of OVs in Co_3_O_4_ material [46, 47]. As we know, OVs are inevitably generated during the deposition of Co_3_O_4_ film under low oxygen pressure. The XPS spectra of O 1*s* are shown in Fig. 4c, and two peaks can be clearly identified. In detail, the peak at 529.3 eV can be deemed the metal–oxygen bonds (i.e., Co–O), while the peak at the high value of 531.2 eV can be regarded as the defect sites with low oxygen coordination (i.e., OV) [46, 48]. Compared with the HRS sample, the intensity of OV peak is obviously strengthened in LRS sample, suggesting that extra OVs can be generated in the LRS sample by electrical field treatment [49].

It is reported that the EPR measurement can provide strong evidence for exploring the existence and variation of OVs [50]. As shown in Fig. 4d, both HRS and LRS samples display an EPR signal at *g* = 2.004, indicating that the OVs exist in both of them [51]. Moreover, the signal intensity of the LRS samples is higher than that of the HRS samples, suggesting more OVs in the LRS sample, which is consistent with the results of XPS. Additionally, Raman measurement is exploited to investigate the OVs of the samples. The Raman spectrum of HRS sample exhibits five distinct characteristic peaks at 195, 479, 520, 616, and 688 cm^−1^, corresponding to the F_2g_^(1)^, E_g_, F_2g_^(2)^, F_2g_^(3)^, and A_1g_ modes of Co_3_O_4_ (Fig. 4e) [52, 53]. Compared with HRS samples, the A_1g_ peak of LRS sample shows a negative shift of 5 cm^−1^, which can be ascribed to the more OVs in LRS sample.

In order to investigate the influence of electric field treatment on the surface electrode, the surface of Ni electrode is characterized by XPS in Fig. 4f. As for the HRS sample, the two peaks located at 852.4 and 869.5 eV are assigned to 2*p*_3/2_ and 2*p*_1/2_ of metallic Ni, respectively, suggesting that the surface of the top electrode in HRS sample is metallic Ni. Different from the HRS sample, after the application of electric field, the peaks attributed to Ni^2+^ 2*p*_3/2_ (855.1 eV) and Ni^2+^ 2*p*_1/2_ (872.2 eV) with the corresponding satellite peaks (860.8 and 879.3 eV) can be also observed on the surface of LRS sample except for the peaks assigned to the metallic Ni. Obviously, the surface Ni electrode is partially oxidized to nickel oxide after the treatment of electric field, which is beneficial to improve the catalytic activity.

### Catalytic Performance of the Samples

**Fig. 5:**
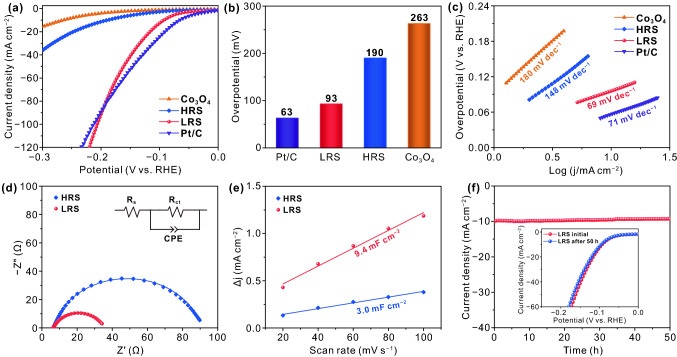
**a** Polarization curves, **b** overpotential and **c** Tafel plots of commercial Pt/C, Co_3_O_4_, HRS sample and LRS sample in 1 M KOH. Scan rate: 10 mV s^−1^. **d** Nyquist plots of HRS sample and LRS sample. **e** Plots of the capacitive currents in CV as a function of the scan rates for HRS sample and LRS sample. The lines show the linear fitting of the plots. **f** Long-term chronopotentiometric stability test of the LRS sample at − 10 mA cm^−2^; inset is the polarization curves of the LRS electrode before and after 50 h for HER

The electrocatalytic activities of the prepared catalysts for HER and OER are investigated through a typical three-electrode system in 1 M KOH solution. Polarization curves are obtained from LSV measurements with a sweep rate of 10 mV s^−1^, which is shown in Fig. 5a. Here all the LSV curves are corrected with iR compensation. Moreover, the commercial Pt/C and RuO_2_ are investigated in the same conditions for further comparison. As shown in Fig. 5b, the pure Co_3_O_4_ requires a large overpotential of 263 mV for HER at 10 mA cm^−2^ due to the lack of H adsorption sites and the low conductivity. In contrast, HRS sample has a smaller overpotential of 190 mV for HER, suggesting that the deposition of Ni metal on Co_3_O_4_ film is beneficial to enhance the electrocatalytic activity by adding the H adsorption sites and improving surface charge transport capability [54, 55]. Strikingly, after the treatment of electric field, LRS sample exhibits a remarkably high catalytic activity with the smallest overpotential of 93 mV to achieve 10 mA cm^−2^ for HER due to the improved electrical conductivity and catalytic activity, which is comparable to the performance of commercial Pt/C (63 mV). It is worth noting that the overpotential of the LRS sample is superior to that of the Pt/C catalyst at high-current density (86 mA cm^−2^ for HER), indicating its high activity and potential application at high-current density. To the best of our knowledge, the overpotential of LRS sample surpasses that of most reported values for cobalt oxide materials in alkaline solution (Table S1). Tafel slope is a crucial metric for electrocatalysts to investigate the electrocatalytic kinetics of HER or OER. As shown in Fig. 5c, LRS sample possesses the lowest Tafel slope of 69 mV dec^−1^ for HER, which is significantly smaller than that of HRS sample (148 mV dec^−1^), Co_3_O_4_ (180 mV dec^−1^) and Pt/C (71 mV dec^−1^), implying the more favorable electrocatalytic reaction kinetics of LRS sample. After the treatment of electric field, the conductive filaments consisting of OVs are formed in the Co_3_O_4_ film, which improves the conductivity of the system and facilitates charge transfer. Meanwhile, the partial oxidation of Ni metal is beneficial to improve HER activity [56, 57]. The aforementioned results indicate that the treatment of electric field can remarkably enhance the HER performance of Ni/Co_3_O_4_ electrocatalyst.

EIS measurements are carried out at − 0.1 V vs. RHE in 1.0 M KOH to study the kinetic activities of the as-obtained catalysts. Figure 5d shows that the charge transfer resistance (*R*_ct_) of LRS sample is obviously lower than that of HRS sample, suggesting the better electrical conductivity and faster charge transport of LRS sample with conductive filaments. To unravel more insight into the influence of electric field treatment on active sites, we investigate the electrochemically active surface area (ECSA) of HRS and LRS samples. The ECSA is linearly proportional to electrochemical double-layer capacitances (*C*_dl_), which can be calculated from the cyclic voltammogram without obvious electrochemical reactions [58, 59]. The *C*_dl_ can be related to the double-layer charging current (*i*_c_) and the scan rate (ν) according to the following equation: *C*_dl_ = *i*_c_/ν [60]. According to the CV curves recorded at the scan rates of 20, 40, 60, 80, and 100 mV s^−1^ (Fig. S3), the plot of scan rate against current density exhibits a good linear relationship, which is shown in Fig. 5e. Obviously, LRS sample shows a larger *C*_dl_ value of 9.4 mF cm^−2^ than that of HRS sample (3.0 mF cm^−2^), implying that it possesses a larger ECSA and more catalytic active sites. The ECSA can be calculated from the *C*_dl_ through the equation: ECSA = *C*_dl_/*C*_s_, where *C*_s_ is the specific capacitance for a flat surface and the typical reported value is 0.040 mF cm^−2^ in 1 M NaOH [60, 61]. As a result, the ECSA of the LRS sample is calculated to be 235 cm^−2^_ECSA_, which is greater than that of HRS sample (75 cm^−2^_ECSA_). Here, the large ECSA is ascribed to the partial oxidation of Ni metal after electric field treatment. Besides, the long-term stability is a crucial index for the practical application of electrocatalysts. The stability test of the LRS sample is conducted in alkaline media through the chronopotentiometric method. As shown in Fig. 5f, the current density of − 10 mA cm^−2^ is rather stable with negligible decay over 50 h and the polarization curve after 50 h is almost coincident with the initial one (Fig. 5f, inset), indicating the extraordinary electrochemical stability of LRS sample for HER. The excellent stability after the treatment of electric field provides good conditions for practical application.

**Fig. 6:**
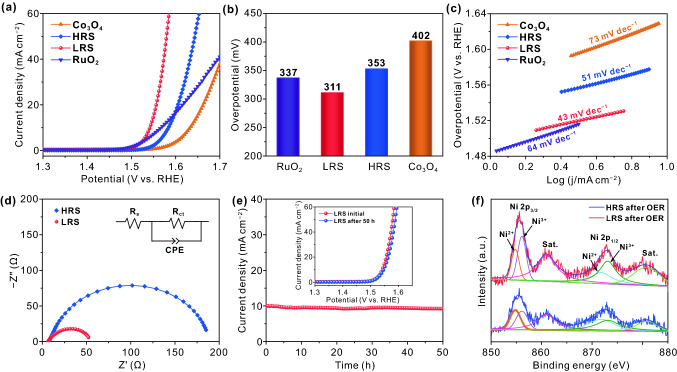
**a** Polarization curves, **b** overpotential, and **c** corresponding Tafel plots of commercial RuO_2_, Co_3_O_4_, HRS sample and LRS sample in 1 M KOH. Scan rate: 10 mV s^−1^. **d** Nyquist plots of HRS sample and LRS sample. **e** The long-term stability of LRS sample under a current density of 10 mA cm^−2^, and its polarization curves (inset) before and after 50 h for OER. **f** XPS spectra of Ni 2*p* for LRS sample and HRS sample after OER operation

In addition to the excellent HER performance, the OER performance is also enhanced after the treatment of electric field in the same electrolyte. The OER electrocatalytic activities of LRS sample, HRS sample, Co_3_O_4_, and commercial RuO_2_ are evaluated in 1.0 M KOH. The LRS sample exhibits the highest electrocatalytic activity with the lowest overpotential of 311 mV to achieve a current density of 10 mA cm^−2^, which is markedly smaller than that of HRS sample (353 mV), Co_3_O_4_ (402 mV), and commercial RuO_2_ catalysts (337 mV) (Fig. 6a, b). The corresponding Tafel plots of these catalysts are shown in Fig. 6c. Obviously, the LRS sample exhibits the smallest Tafel value of (43 mV dec^−1^), which is superior to that of HRS sample (51 mV dec^−1^), Co_3_O_4_ (73 mV dec^−1^), and commercial RuO_2_ (64 mV dec^−1^), indicating a highly favorable OER kinetic for this catalyst. The OER performance of the LRS sample is comparable to the recently reported Co-based electrocatalysts in Table S2. In addition, the EIS measurement is performed at 1.54 V vs. RHE. As shown in Fig. 6d, the LRS sample possesses the smaller semicircle than that of the HRS sample, revealing its rapid charge transfer kinetics and excellent electron conductivity [62]. Moreover, a chronoamperometry measurement is carried out to investigate the stability of LRS sample. As displayed in Fig. 6e, the LRS sample exhibits a slight current attenuation for a period of 50 h at 10 mA cm^−2^ and the polarization curve after 50 h is almost the same as the initial one (Fig. 6e, inset), indicating the excellent stability of LRS sample for OER. The surface morphology of LRS sample shows the insignificant change before and after OER operation (Fig. S4), which verifies its excellent stability. Moreover, the XPS spectrum of Co 2*p* is measured in bulk for LRS sample after OER operation (Fig. S5). The value of Co^3+^/Co^2+^ of LRS sample after OER operation is 0.86, which is almost equal to the value before reaction (0.85, Fig. 4b). Thus, the structure of bulk remains unchanged during the OER. In order to investigate the influence of OER operation on the catalyst surface, XPS characterization is performed on the surface Ni electrodes of HRS sample and LRS sample after OER operation. It can be found in Fig. 6f that the Ni^2+^/Ni^3+^ peaks exist in both HRS and LRS samples, indicating that the surface Ni metal has been oxidized after the OER reaction. According to XPS results, the value of Ni^3+^/Ni^2+^ of the LRS sample is 1.62, which is greater than that of the HRS sample (1.08). According to the earlier reports, Ni^3+^ is active for OER, suggesting that LRS sample with more Ni^3+^ exhibits superior OER activity [63–65]. Figure S6 shows the XPS spectra of Co 2*p* on the Co_3_O_4_ surface of LRS sample before and after OER test. It can be observed that the value of Co^3+^/Co^2+^ after OER test is greater than that before reaction, suggesting that Co^3+^ atoms on the surface are the main active sites in Co_3_O_4_ material [66, 67].

**Fig. 7:**
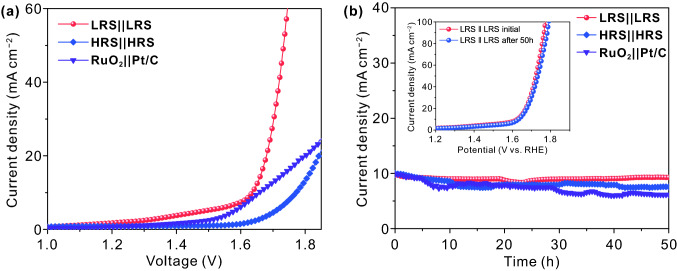
**a** Polarization curves and **b** the long-term stability of LRS ǁ LRS couple, HRS ǁ HRS couple and RuO_2_ ǁ Pt/C couple under a current density of 10 mA cm.^−2^; inset is the polarization curves of LRS ǁ LRS couple before and after 50 h for overall water splitting

In view of the excellent bifunctional HER and OER activities, a two-electrode configuration is assembled to investigate the catalytic performance of overall water splitting for the LRS and HRS samples. Meanwhile, the catalytic performance of noble metal RuO_2_ ǁ Pt/C electrocatalyst is investigated either as a control group. It is found that the LRS ǁ LRS couple is capable of affording a current density of 10 mA cm^−2^ at a voltage of 1.63 V (Fig. 7a), which is lower than that of the coupled RuO_2_ ǁ Pt/C catalyst (1.66 V) and the HRS ǁ HRS couple (1.77 V). Especially for high-current density, such as 20 mA cm^−2^, the advantage of LRS ǁ LRS couple (1.68 V) is more significant, which is markedly lower than that of the coupled RuO_2_ ǁ Pt/C catalyst (1.80 V) and the HRS ǁ HRS couple (1.84 V). The excellent performance of LRS ǁ LRS couple is comparable to the most reported bifunctional electrocatalysts (Table S3). In addition, the long-term operating performance of the above bifunctional electrocatalysts is probed at 10 mA cm^−2^. As shown in Fig. 7b, compared with the other two electrocatalysts, the LRS ǁ LRS couple exhibits the lightest attenuation during the 50 h chronoamperometry test, indicating that the LRS ǁ LRS couple has an excellent long-term stability. Moreover, the polarization curve of LRS ǁ LRS couple after 50 h exhibits an almost negligible change compared with the initial one (Fig. 7b, inset). These results indicate that the LRS sample possesses an extraordinary electrochemical stability for overall water splitting, which is beneficial to industrial applications.

### Mechanism Analysis of the Enhanced Catalytic Performance

**Fig. 8:**
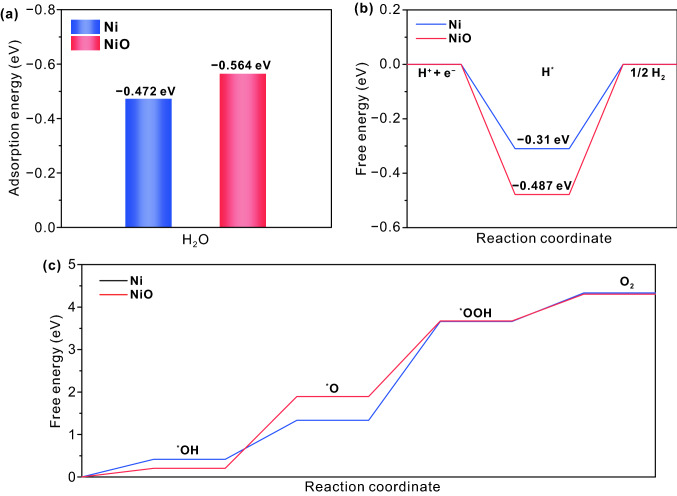
**a** Calculated adsorption energy for water on the surface of Ni and NiO. **b** Calculated free energy of hydrogen adsorption diagram on the surface of Ni and NiO. **c** Calculated OER free energy diagram on the surface of Ni and NiO

Compared with HRS sample, the Ni electrode of LRS sample is partially oxidized to NiO after the electric field treatment. To gain insights into the influence of electric field treatment on HER and OER kinetics, a series of DFT calculations for Ni and NiO are performed. During the process of hydrogen evolution in alkaline solution, water molecules are first adsorbed on the catalyst surface, and then dissociated to form H_2_. Therefore, the adsorption energy of H_2_O and the Gibbs free energy of hydrogen adsorption (Δ*G*_H_) of NiO and Ni are calculated for the HER. Figure 8a shows that the adsorption energy of NiO (− 0.564 eV) for H_2_O is greater than that of Ni (− 0.472 eV), suggesting that water is more easily adsorbed on NiO surface. In addition, the ΔG_H_ of Ni (− 0.31 eV) is smaller than that of NiO (− 0.487 eV) and closer to the optimal value (Fig. 8b), which indicates that the conversion of H* to H_2_ is much easier on the Ni surface. Therefore, the synergy between Ni and NiO promotes the HER kinetics.

According to the four-electron mechanism for the OER [68], the adsorption free energy of O-containing intermediates on Ni and NiO in alkali media is calculated. As shown in Fig. 8c, the OER rate determining step is the formation of *OOH group from *O group. Moreover, the free energy between *O and *OOH of NiO (1.78 eV) is smaller than that of Ni (2.33 eV), revealing a faster OER kinetics of NiO. In addition, this conclusion can be demonstrated by more Ni^3+^ on the surface of LRS samples after OER operation in Fig. 6f. Therefore, the oxygen evolution catalyzed by NiO is more favorable than Ni.

**Fig. 9:**
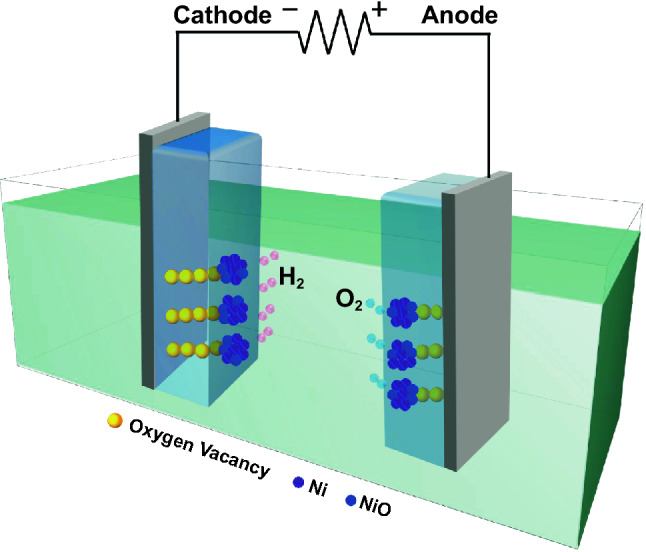
Schematic diagram of the mechanism of LRS sample as a bifunctional electrocatalyst for overall water splitting

Based on the abovementioned analysis, we can explore the origin of excellent electrocatalytic performance for LRS sample. The schematic diagram of the preliminary mechanism of the LRS catalyst for overall water splitting is illustrated in Fig. 9. Compared with the HRS sample, the Ni metal is partially oxidized to nickel oxide after the treatment of electric field, which enlarges the electrochemical surface area and increases the number of active sites in the LRS sample. According to the DFT calculations, NiO is beneficial to the adsorption of water, while Ni is easier to release H_2_. Therefore, the synergistic effect of NiO and Ni improves the HER activity. Meanwhile, DFT calculated results also suggest that NiO is favorable for the formation of *O to *OOH, revealing a superior OER activity. More importantly, under the action of an external electric field, the OVs in Co_3_O_4_ film align along the electric field direction and form some conductive filaments in bulk, which significantly reduces the system resistivity and accelerates the charge transport. Under the synergistic effect of the above favorable conditions, the LRS sample as a bifunctional electrocatalyst achieves an excellent electrocatalytic performance.

## Conclusions

Co_3_O_4_ film and Ni electrode are deposited on the substrate sequentially by PLD in this work. The strategy of electric field treatment is adopted to form stable conductive filaments consisting of OVs in the oxide film, which can convert the sample from HRS to LRS and improve the electrical conductivity of system. In addition, under the action of electric field, the Ni metal is partially oxidized to nickel oxide, which increases the number of active sites and enhances the catalytic activity. Therefore, as a bifunctional electrocatalyst, the LRS sample possesses excellent HER and OER performance, and can effectively catalyze overall water splitting when used directly as an anode and cathode catalyst. Moreover, the nonvolatile characteristic of the conductive filaments in bulk makes the LRS sample possess extraordinary long-term stability, which provides conditions for industrial production. We believe that the knowledge gained in this work can provide new ideas and methods for the rational design of catalysts in a wide range of applications.

## Supplementary Information

Below is the link to the electronic supplementary material.

Supplementary file1 (PDF 772 kb)
