# Supplementary material for: Electric-Field-Treated Ni/Co3O4 Film as High-Performance Bifunctional Electrocatalysts for Efficient Overall Water Splitting
Source: Nanomicro Lett. 2022 Jul 22;14:148. doi: 10.1007/s40820-022-00889-3 (PMC9307702; doi:10.1007/s40820-022-00889-3)
Supplement: Supplementary file 1 — Supplementary file1 (PDF 772 kb) [file 40820_2022_889_MOESM1_ESM.pdf]

Supporting Information for

Electric-Field-Treated Ni/Co_3O_4 Film as High-Performance Bifunctional Electrocatalysts for Efficient Overall Water Splitting

Junming Li[1], Jun Li[1], Jun Ren[1], Hong Hong[1], Dongxue Liu[1], Lizhe Liu[1], Dunhui Wang[1, 2, *]

[1]National Laboratory of Solid State Microstructures, Collaborative Innovation Center of Advanced Microstructures, Jiangsu Provincial Key Laboratory for Nanotechnology, School of Physics, Nanjing University, Nanjing 210093, P. R. China

[2]Hangzhou Dianzi University, Hangzhou 310018, P. R. China

*Corresponding author. E-mail: wangdh@hdu.edu.cn (Dunhui Wang)

Supplementary Figures and Tables

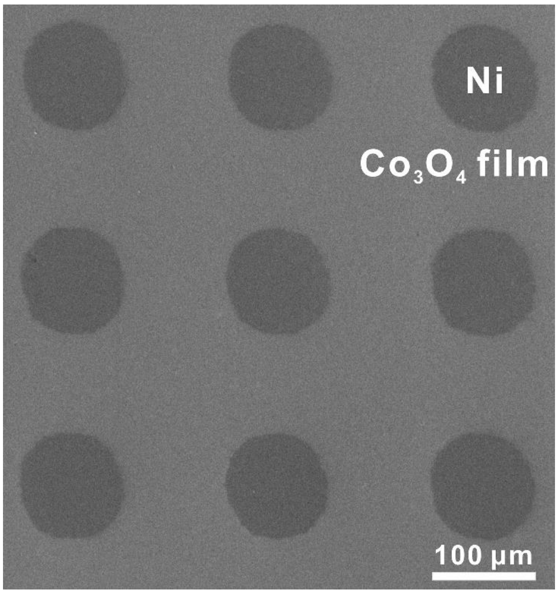

Fig. S1 The surface SEM image of the Ni/Co_3O_4/Pt device

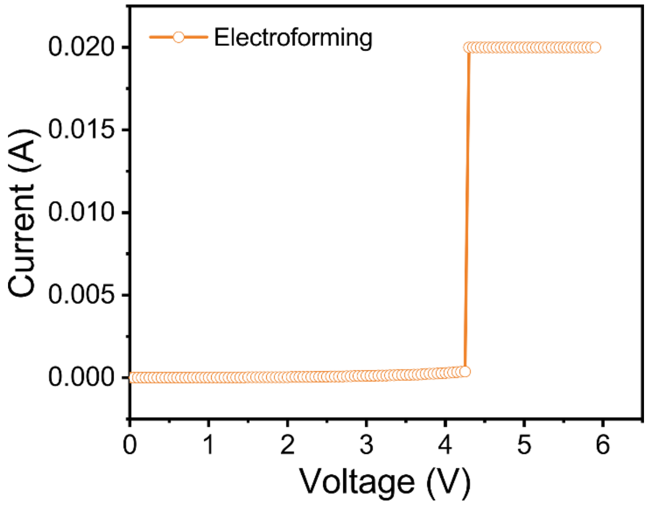

Fig. S2 The electroforming process of the prepared device. The current compliance (I_{cc}) is 0.02 A

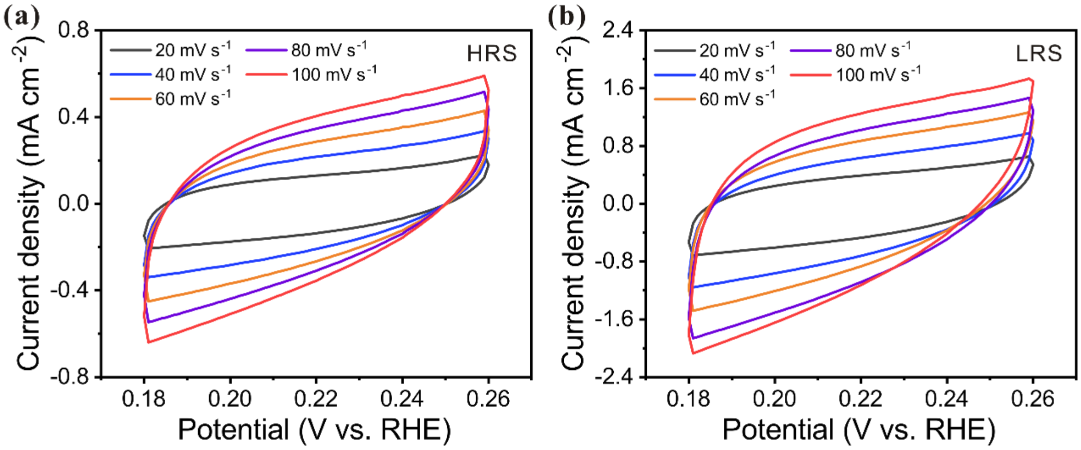

Fig. S3 Electrochemical surface area (ECSA) tests of different samples. Cyclic voltammogram curves of (**a**) HRS sample and (**b**) LRS sample measured in the nonfaradaic potential of 0.18-0.26 V vs. RHE at multiple scan rates (20, 40, 60, 80 and 100 mV s^{-1})

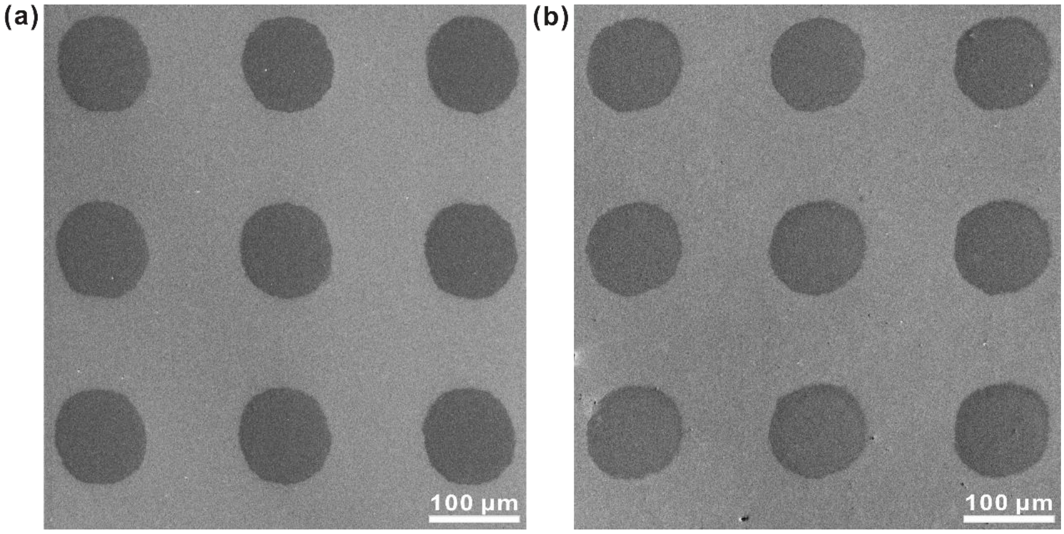

Fig. S4 The surface SEM image of LRS sample before and after OER text

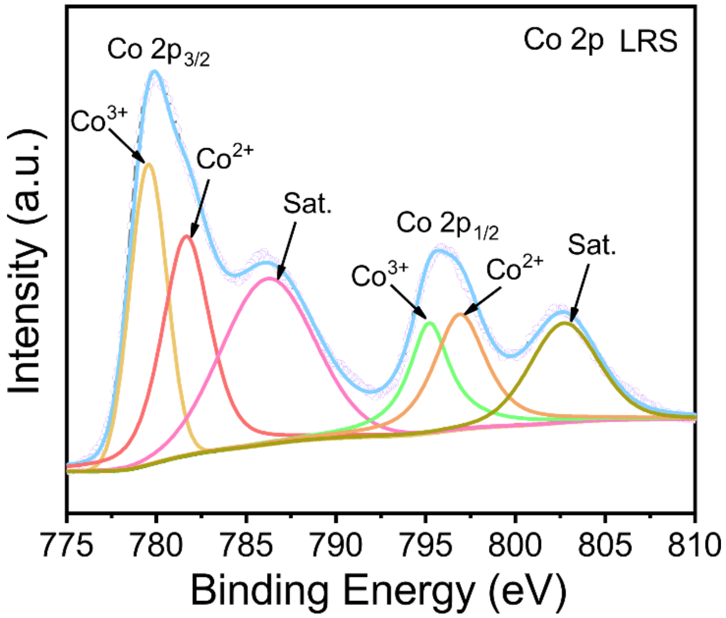

Fig. S5 XPS spectra of Co 2p in bulk for LRS sample after OER operation

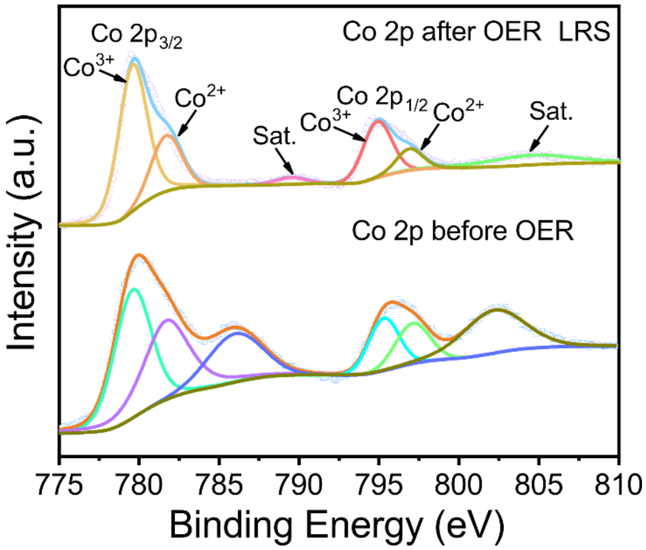

Fig. S6 XPS spectra of Co 2p on the surface of LRS sample before and after OER test

Table S1 Comparison of HER catalytic performance of LRS sample with other reported cobalt-based catalysts in alkaline electrolyte

Catalyst	Electrolyte	Current density (*j*)	Overpotential (mV)	Tafel (mV dec^{-1})	Refs.
**LRS sample**	**1M KOH**	**10**	**93**	**69**	**This work**
Ni/Co_3O_4 cubes	1 M KOH	10	145	109	[S1]
NiO/Co_3O_4 microcubes	1 M KOH	10	169.5	119	[S2]
MOF Co_3O_4/MoS_2	1 M KOH	10	205	98	[S3]
Cu-Co_3O_4 nanoarrays	1 M KOH	10	125	66	[S4]
$P_{8.6}$-Co_3O_4/NF	1 M KOH	10	97	86	[S5]
Co_3O_4-N-C	1 M KOH	10	139	45	[S6]
P-Co_3O_4	1 M KOH	10	120	51.6	[S7]
Co_3O_4@Ni	1 M KOH	10	225	53	[S8]
Co_3O_4 with {111} crystal planes	1 M KOH	10	195	50	[S9]
Co_3O_4 NCs/C	1 M KOH	10	380	116	[S10]

Table S2 Comparison of OER catalytic performance of LRS sample with other reported cobalt-based catalysts in alkaline electrolyte

Catalyst	Electrolyte	Current density (j)	Overpotential (mV)	Tafel (mV dec^{-1})	Refs.
LRS sample	1 M KOH	10	311	43	This work
Ni/Co_3O_4 cubes	1 M KOH	10	324	35	[S1]
Co_3O_4/$CoMoO_4$	1 M KOH	10	318	63	[S11]
Co_3O_4 NS	1 M KOH	10	330	57.8	[S12]
Co_3O_4 nanowires	1 M KOH	10	339	82	[S13]
Co_3O_4/ $NiCo_2O_4$ nanocages	1 M KOH	10	340	88	[S14]
Co_3O_4 films	1 M NaOH	10	377	58.1	[S15]
Mesoporous Co_3O_4	1 M KOH	10	380	59	[S16]
Co_3O_4 NCs/C	1 M KOH	10	320	101	[S10]
Meso-Co_3O_4 NWs	1 M KOH	10	370	72	[S17]
Co_3O_4/N-rmGO	1 M KOH	10	310	67	[S18]

Table S3 Comparison of overall water splitting performance of LRS sample with other reported cobalt-based bifunctional electrocatalysts in alkaline solution

**Catalyst**	**Electrolyte**	**Current density (j)**	**Potential (V)**	**Refs.**
LRS sample	1 M KOH	10	1.63	This work
Ni/Co_3O_4/Ti-mesh	1 M KOH	10	1.66	[S1]
$P_{8.6}$-Co_3O_4/NF	1 M KOH	10	1.63	[S5]
Co_3O_4-MTA	1 M KOH	10	1.63	[S19]
Co_3O_4@Ni	1 M KOH	10	1.64	[S8]
RuO_2/Co_3O_4	1 M KOH	10	1.645	[S20]
Co_3O_4 NCs/C	1 M KOH	10	1.91	[S10]
$NiCo_2O_4$ microcuboids	1 M KOH	10	1.65	[S21]
CoO-CNF	1 M KOH	10	1.63	[S22]
$Co_5Mo_{1.0}O$ NSs@NF	1 M KOH	10	1.68	[S23]
$NiCo_2S_4$ NA/CC	1 M KOH	10	1.68	[S24]

Supplementary References

[S1] M.S. Riaz, S. Zhao, C. Dong, M.J. Iqbal, Y. Zhao et al., Spherical sacrificial ZnO template-derived hybrid Ni/Co_3O_4 cubes as efficient bifunctional electrocatalyst for overall water splitting. Energy Technol. **8**(5), 1901310 (2020). https://doi.org/10.1002/ente.201901310

[S2] X. Wei, Y. Zhang, H. He, D. Gao, J. Hu et al., Carbon-incorporated NiO/Co_3O_4 concave surface microcubes derived from a MOF precursor for overall water splitting. Chem. Commun. **55**(46), 6515-6518 (2019). https://doi.org/10.1039/c9cc02037c

[S3] A. Muthurasu, V. Maruthapandian, H.Y. Kim, Metal-organic framework derived Co_3O_4/MoS_2 heterostructure for efficient bifunctional electrocatalysts for oxygen evolution reaction and hydrogen evolution reaction. Appl. Catal. B **248**, 202-210 (2019). https://doi.org/10.1016/j.apcatb.2019.02.014

[S4] Y. Tian, L. Cao, P. Qin, Bimetal-organic framework derived high-valence-state Cu-doped Co_3O_4 porous nanosheet arrays for efficient oxygen evolution and water splitting. ChemCatChem **11**(17), 4420-4426 (2019). https://doi.org/10.1002/cctc.201900834

[S5] Z. Wang, H. Liu, R. Ge, X. Ren, J. Ren et al., Phosphorus-doped Co_3O_4 nanowire array: a highly efficient bifunctional electrocatalyst for overall water splitting. ACS Catal. **8**(3), 2236-2241 (2018). https://doi.org/10.1021/acscatal.7b03594

[S6] B. You, Y. Zhang, P. Yin, D. Jiang, Y. Sun, Universal molecular-confined synthesis of interconnected porous metal oxides-N-C frameworks for electrocatalytic water splitting. Nano Energy **48**, 600-606 (2018). https://doi.org/10.1016/j.nanoen.2018.04.009

[S7] Z. Xiao, Y. Wang, Y. Huang, Z. Wei, C. Dong et al., Filling the oxygen vacancies in Co_3O_4 with phosphorus: an ultra-efficient electrocatalyst for overall water splitting. Energy Environ. Sci. **10**(12), 2563-2569 (2017). https://doi.org/10.1039/c7ee01917c

[S8] R. Li, D. Zhou, J. Luo, W. Xu, J. Li et al., The urchin-like sphere arrays Co_3O_4 as a bifunctional catalyst for hydrogen evolution reaction and oxygen evolution reaction. J. Power Sources **341**, 250-256 (2017). https://doi.org/10.1016/j.jpowsour.2016.10.096

[S9] L. Liu, Z. Jiang, L. Fang, H. Xu, H. Zhang et al., Probing the crystal plane effect of Co_3O_4 for enhanced electrocatalytic performance toward efficient overall water

splitting. ACS Appl. Mater. Interfaces **9**(33), 27736-27744 (2017). https://doi.org/10.1021/acsami.7b07793

[S10] S. Du, Z. Ren, J. Zhang, J. Wu, W. Xi et al., Co_3O_4 nanocrystal ink printed on carbon fiber paper as a large-area electrode for electrochemical water splitting. Chem. Commun. **51**(38), 8066-8069 (2015). https://doi.org/10.1039/c5cc01080b

[S11] L. Zhang, T. Mi, M.A. Ziaee, L. Liang, R. Wang, Hollow POM@MOF hybrid-derived porous $Co_3O_4/CoMoO_4$ nanocages for enhanced electrocatalytic water oxidation. J. Mater. Chem. A **6**(4), 1639-1647 (2018). https://doi.org/10.1039/c7ta08683k

[S12] G. Zhang, J. Yang, H. Wang, H. Chen, J. Yang et al., $Co_3O_{4-\delta}$ quantum dots as a highly efficient oxygen evolution reaction catalyst for water splitting. ACS Appl. Mater. Interfaces **9**(19), 16159-16167 (2017). https://doi.org/10.1021/acsami.7b01591

[S13] Y. Zhang, B. Ouyang, J. Xu, G. Jia, S. Chen et al., Rapid synthesis of cobalt nitride nanowires: highly efficient and low-cost catalysts for oxygen evolution. Angew. Chem. Int. Ed. **55**(30), 8670-8674 (2016). https://doi.org/10.1002/anie.201604372

[S14] H. Hu, B. Guan, B. Xia, X.W. Lou, Designed formation of $Co_3O_4/NiCo_2O_4$ double-shelled nanocages with enhanced pseudocapacitive and electrocatalytic properties. J. Am. Chem. Soc. **137**(16), 5590-5595 (2015). https://doi.org/10.1021/jacs.5b02465

[S15] H.S. Jeon, M.S. Jee, H. Kim, S.J. Ahn, Y.J. Hwang et al., Simple chemical solution deposition of Co_3O_4 thin film electrocatalyst for oxygen evolution reaction. ACS Appl. Mater. Interfaces **7**(44), 24550-24555 (2015). https://doi.org/10.1021/acsami.5b06189

[S16] S. Chen, Y. Zhao, B. Sun, Z. Ao, X. Xie et al., Microwave-assisted synthesis of mesoporous Co_3O_4 nanoflakes for applications in lithium ion batteries and oxygen evolution reactions. ACS Appl. Mater. Interfaces **7**(5), 3306-3313 (2015). https://doi.org/10.1021/am508136k

[S17] Y. Wang, T. Zhou, K. Jiang, P. Da, Z. Peng et al., Reduced mesoporous Co_3O_4 nanowires as efficient water oxidation electrocatalysts and supercapacitor electrodes. Adv. Energy Mater. **4**(16), 1400696 (2014). https://doi.org/10.1002/aenm.201400696

[S18] Y. Liang, Y. Li, H. Wang, J. Zhou, J. Wang et al., Co_3O_4 nanocrystals on graphene as a synergistic catalyst for oxygen reduction reaction. Nat. Mater. **10**(10), 780-786 (2011). https://doi.org/10.1038/nmat3087

[S19] Y.P. Zhu, T.Y. Ma, M. Jaroniec, S.Z. Qiao, Self-templating synthesis of hollow Co_3O_4 microtube arrays for highly efficient water electrolysis. Angew. Chem. Int. Ed. **56**(5), 1324-1328 (2017). https://doi.org/10.1002/anie.201610413

[S20] H. Liu, G. Xia, R. Zhang, P. Jiang, J. Chen et al., MOF-derived RuO_2/Co_3O_4 heterojunctions as highly efficient bifunctional electrocatalysts for HER and OER in alkaline solutions. RSC Adv. **7**(7), 3686-3694 (2017). https://doi.org/10.1039/c6ra25810g

[S21] X. Gao, H. Zhang, Q. Li, X. Yu, Z. Hong et al., Hierarchical $NiCo_2O_4$ hollow microcuboids as bifunctional electrocatalysts for overall water-splitting. Angew. Chem. Int. Ed. **55**(21), 6290-6294 (2016). https://doi.org/10.1002/anie.201600525

[S22] H. Wang, H. Lee, Y. Deng, Z. Lu, P. Hsu et al., Bifunctional non-noble metal oxide nanoparticle electrocatalysts through lithium-induced conversion for overall water splitting. Nat. Commun. **6**, 7261 (2015). https://doi.org/10.1038/ncomms8261

[S23] Y. Zhang, Q. Shao, S. Long, X. Huang, Cobalt-molybdenum nanosheet arrays as highly efficient and stable earth-abundant electrocatalysts for overall water splitting. Nano Energy **45**, 448-455 (2018). https://doi.org/10.1016/j.nanoen.2018.01.022

[S24] D. Liu, Q. Lu, Y. Luo, X. Sun, A.M. Asiri, $NiCo_2S_4$ nanowires array as an efficient bifunctional electrocatalyst for full water splitting with superior activity. Nanoscale **7**(37), 15122-15126 (2015). https://doi.org/10.1039/c5nr04064g
